# An overview of civil society organizations’ roles in health project sustainability in Bauchi State, Nigeria

**DOI:** 10.11604/pamj.2018.30.150.15851

**Published:** 2018-06-20

**Authors:** Umar Ibrahim, Sharifa Ezat Wan-Puteh

**Affiliations:** 1 Department of Community Health, Faculty of Medicine, University of Kebangsaan, Bandar,Tun Razak, Jalan Yacoob Latif, 56000 Cheras, Malaysia

**Keywords:** CSOs, health, project, sustainability, governance, partnership, resources, community

## Abstract

**Introduction:**

This study sets out to assess the roles of Civil Society Organizations (CSOs) in post donor health project sustainability in Low and Middle Income Countries (LMICs), the case of the Bauchi State, Nigeria. This study equally investigated the CSOs strategies and roles in health project sustainability.

**Methods:**

For quantitative data collection, the random, purposive, and convenient sampling techniques were used and 156 respondents selected from relevant organizations operating in Bauchi state, Nigeria, and 15 respondents for Key Informant Interviews (KIIs). A Semi-structured questionnaire was the study instrument, and consent from the participants as well as ethical clearances were duly obtained.

**Results:**

The study revealed that 87.8% of the respondents indicate un-friendly operational policies, while 88.9% of them identified lack of resources (human, money and machineries) as impediments to project sustainability. Also, 74.3% of the respondents said partnership among key stakeholders and 86.6% of them affirmed that community participation and use of available (local) resources ensure health project sustainability. The study further revealed that CSOs fund health projects, support government efforts and encourage development of project sustainability road map in the state.

**Conclusion:**

Hence, health project sustainability plan should form part of a project right from inception through the donor period and thereafter. In addition to the above, internal income framework, community involvement, enabling policies and partnership among stakeholders, especially with the host government, should always guide project implementation, because without these in place, project sustainability will remain a mirage.

with public sectors, usually sponsored by external donors. These donations emerge from different organizations, some of which are multilateral, like the UN (United Nations), the WHO (World Health Organizations) etc, bilateral like: USAID (United State Agency for International Development), Global Health Initiatives such as Bill and Melinda Gates Initiative (BMGI) and Global Fund to fight Malaria, Tuberculosis and HIV/AIDS (GFAM), International Non-Governmental Organizations (INGO) like: Oxfam and PLAN and other plethora of donor organizations. Availability of external funding sustains a project but the project stops when the funding is no longer available. This situation affects the sustainability of the project. In practical terms, most health projects received almost no attention in post-donor era, indicating an obvious lack of concern for project sustainability issues. As such, health project sustainability mechanism is literally the ’life-line‚ of a project. In this study, health project sustainability as a concept denotes project continuity in terms of quality, availability and accessibility at the end of donor intervention. Sustainability refers to the activities that ensure project capacity maintenance after donor support ends, preventing it from being phased out [2, 3]. CSOs as key partners in development cooperation conduct activities that ensure project sustainability, these activities include, but not limited to policy support, strategic planning, service implementation, project evaluation etc [4]. Roles of CSOs in health project sustainability are mainly complimentary in support of government efforts [5]. But, the nature of their activities varies, depending on the country and issue in contention. These roles are however not without challenges, in need of support with human and capital resources [6, 7]. The Bauchi state, Nigeria, was selected for the study because it represents the country's health project landscape, due to the presence of several donor supported health projects in the state, performing diverse roles. Despite their activities in the state, there exists limited literature on their roles in health project sustainability in the state, considering the growing interest on Sustainable Development Goals (SDGs) implementation by UN member states. Against this background, this study assesses the roles of CSOs in project sustainability, to provide policy and decision makers with evidence-based information that can be applied in developing sustainable approaches that ensure health project continuity after donors exit.

## Methods

Bauchi State, Nigeria, was the study area. The state is located in the north of equator at latitude 903' and 1203', and longitudinally between 805I and 110, east of the Greenwich meridian [8]. The State has a population figure of 4,676,465, with density of 95 people per square kilometers according to the 2006 population census [9]. The state is endowed with all the characteristics of Low and Middle Income Countries, and there are several stakeholders in the health system strengthening endeavors, which make the state an ideal setting for the study. This study employed a descriptive field survey approach through which roles of CSOs in health project sustainability were explored. The study synthesized responses obtained from 156 respondents drawn through random sampling from a list of CSOs operating in the state, obtained from Bauchi State Network of Civil Society Organization (BASNEC). A purposive sampling technique was used to select respondents from government and multilateral agencies, while a convenient sampling method was used to select individual respondents and private sector actors, including the 15 Key Informant Interviews' respondents (KIIs). The KIIs' respondents are those with experience in dealing with CSOs and also occupying leadership positions in the Rank of Directors, Head of Departments, Civil Society Advisors, Trustees, Executive Board members, and Program officers respectively. A semi-structured questionnaire was the study instrument, which was based on a 5 point likert scale options from strongly disagree, disagree, undecided, agree and strongly agree responses, distributed by the researcher and a trained research assistant. The questionnaire was examined and validated by experienced health researchers at Abubakar Tafawa Balewa University, Bauchi; and University of Kebangsaan, Malaysia. Cronbach's Alpha reliability of the instrument was 0.89, indicating high content reliability. The purpose of this study was fully explained to the respondents, confidentiality ensured, informed consent and ethical approval were all obtained. The collected data were analyzed using descriptive statistic (frequency and percentage) in SPSS version 21. The qualitative method utilized Key Informant Interview (KII) technique, to explore the issues under study which evolve from two main questions, thus; what are the health CSOs contributions to sustainability of health project in the state?, and what can health system CSOs do to ensure health project sustainability in the state?

## Results

Table 1 shows the distribution of 156 respondents of the study drawn from seven Civil Society Organizations (CSOs) in Bauchi state, Nigeria. The table reveals in proportion the types of organizations involved in the study, and a majority of the respondent (41%) is from the Non-Governmental organizations (NGOs). In Table 2, 51.3% (n=80) of the respondents confirmed that sustainability mechanism is a criterion used by donors to ensure continuity of health programs. Also, 56.4% (n=88) and 25.6% (n=40) agreed and strongly agreed that understanding of a project's pain points ensures sustainability. Also, 42.9% (n=67) of the respondents agreed, while 12.8% disagreed with the statement that sustainable management is grounded on partnership with stakeholders. The findings indicated that 49.4% of the respondents strongly agreed that community participation ensures project sustainability. In a similar manner, 53.8 % (n=84) of the respondents agreed with the statement that uneven work load among staff challenge project sustainability. The results in Table 3indicate that 88.9% of the respondents agreed that sustainability requires sufficient resources to maintain quality, 53.8% supported the claim that sustainability should be tailored towards local needs and available resources. On the assumptions that interventions are required to achieve sustainable outcomes, 51.3% of the respondents agreed. From the study, 16% were undecided on the statement that CSOs dependency on donor funding impede sustainability. The findings revealed that majority of the respondents (87.8%) believed that lack of operation friendly policies hinder program sustainability.

**Table 1:** Types of organizations

Distribution	frequency	percentage
Government/Multilateral organizations	28	17.9
Non-Governmental Organizations (NGOs)	65	41.0
Trade Union	8	5.1
Faith Based Organization (FBOs)	16	10.3
Private Sector	16	10.3
Individual	8	5.1
Community Based Organizations (CBOs)	16	10.3
**Total**	100	156

**Table 2:** Understanding project sustainability

Statement	Options % (N)				
	1	2	3	4	5
Sustainability mechanism is a criterion used by donors to ensure continuity of health programs	1.3 (2)	3.8 (6)	9.0 (14)	51.3 (80)	34.6 (54)
Understanding of a project’s pain points ensures sustainability	0.6 (1)	3.2 (5)	14.1 (22)	56.4 (88)	25.6 (40)
Sustainable management is grounded on partnership with stakeholders	13 (2)	12.8 (20)	11.5 (18)	42.9 (67)	31.4 (67)
Community participation ensures project sustainability	0.6 (1)	3.2 (5)	9.6 (15)	49.4 (77)	37.2 (58)
Uneven work load among staff challenge project sustainability	1.9 (3)	8.3 (13)	10.9 (17)	53.8 (84)	25 (39)

**Key:** 1= strongly disagree; 2 = disagree; 3)undecided; 4= agreed; 5= strongly agreed

**Table 3:** Resources & intervention

Statement	Options % (N)				
	1	2	3	4	5
Sustainability required sufficient resources to maintain quality	2.6(4)	2.6 (4)	6.4 (10)	50.4 (78)	38.5 (60)
Sustainability should be tailored to local needs and available resources	2.6(4)	3.8 (6)	7.1 (11)	53.8 (84)	32.7 (51)
Interventions are required to achieve sustainable outcomes	1.3(2)	3.8 (6)	9.0 (14)	51.3 (80)	34.6 (54)
CSOs dependency on donor funding impede sustainability	1.9(3)	77 (12)	16 (25)	41 (64)	33.3 (52)
Lack of operation friendly policies hinders program sustainability	1.9(3)	1.9 (3)	8.3 (13)	78 (50)	37.8 (59)

**Key:** 1= strongly disagree; 2 =disagree; 3= undecided; 4=agreed; 5=strongly agreed

**Key Informant Interview (KII):** Project sustainability denotes the ability to maintain and keep program functioning in post donor era. CSOs contribute enormously to health project sustainability in many forms, some of which include: funding, policy support, capacity building, community engagements, information dissemination, and advocacy amongst other issues identified by the respondents.

**Policy formulation support:** CSOs support policy formulation that addresses health issues in contention as put in by one respondent. *“They contribute to health policymaking through supplying of information that enhanced program sustainability” (KI 2)*

**Community engagements:** Community engagement is important in ensuring project sustainability, it entails engaging the community at the beginning of a project, middle, end and after. The respondents highlight this importance thus;

“Community participation in a project enables community members to contribute their quota in program implementation” (KI 11); “CSOs help in creation of Community Based Health Volunteers (CBHV) and Ward Development Community (WDC) through community engagement activities” (KI 7); “Feeling of project ownership is developed through community participation in which a motivation to do more manifest” (KI 13); “ Support for immunizations was realized through community engagements” (KI 5).

**Advocacy campaign:** CSOs raise public awareness and disseminate information to the state through advocacy campaign. The respondents identified CSOs contribution to program sustainability through advocacy campaign.

“Through advocacy the CSOs exert pressure on government to provide and maintain treatment accessibility during disease outbreak as well as public sensitization campaign on the disease” (KI 4); “CSOs enlighten the public on importance of monitoring of services provided in their community to encourage transparency and continuity” (KI 6) “CSOs engaged ministry leadership on the need to develop program sustainability roadmap in the state” (KI 15).

**Implementation roles:** According to the respondents, the CSOs implement services that enhance project sustainability; they monitor service provision, generate data through diseases surveillance and also serve as conduit for community reach.

“ They conduct direct service implementation in grassroots' health facilities”(KI 9) “They conduct disease surveillance and reporting activities” (KI 12) “They serve as an entry point to communities by other CSOs” (KI 5) “ The support government during outbreak”(KI 1) “They monitor government program implementation”(KI 10).

**Resource mobilization and development:** CSOs mobilize required resources for effective functioning. The respondents identified human, financial and infrastructure as main resources needed for project sustainability.

“They CSOs fund and trained services providers”(KI 8) “They built health professionals' capacity” (KI 3) ; “They trained TBAs, CBDs and VVHW” (KI 15); “Most of the voluntary health' workers in the state are trained by CSOs” (KI 14).

**Partnership:** Partnership enhances project sustainability. Partnership among the health governance actors; as stipulated by one respondent ensures sustainability. *“Sustainability mechanism lies upon partnership between stakeholders” (KI 2)*.

## Discussion

NGOs emerged as the organization (Table 1) with the majority responses (41%). Role played by CSOs in project sustainability depends upon their type, characteristics and capacity. CSOs possess the ability to influence actions which make them effective as project sustainability agents. Sustainability is a criterion employed by donors to ensure that a project could be sustained in post donor period, and this statement was supported by 85.9% of the respondents (Table 2). Despite the fact that sustainability is a criterion for donor assisted projects, it is usually not adhered to, as evidenced by attention given to a proceeding project, disregarding sustainability outcome of the completed project. Sustainability mechanism as a criterion for project continuity should be adhered to at every stage of project life. Also, understanding a project's pain points ensures sustainability as confirmed by 82% of the respondents (Table 2), which is in line with another study finding which confirmed lack of project understanding by the host community as a pain point [10, 11]. For example, if a program is designed to improve reproductive health during pre-natal care, the aim could be defeated if the target women did not understand what the project is all about and as such, may not make use of the project. Therefore, as a solution, every possible means that ensures project understanding should be extrapolated. Interestingly, 74.3% of the respondents (Table 2) confirmed that sustainable management is grounded on partnership with stakeholders. Indeed, partnership between CSOs and community members or between CSOs and government ensure project sustainability. Appropriate partnership between CSOs, state, development partners, private sectors focusing on the implementation of health project, enhance sustainability commitment towards achieving post donor projects' objectives. The respondents of this study confirmed that community participation ensures project sustainability. Community participation facilitates pre and post project intervention for sustainability. It is a crucial tool that binds stakeholders, and inculcates a sense of belonging to the community. Processes that involved community members enable effective information dissemination among stakeholders, which in turn promote project sustainability [12]. However, community participation alone is not enough; community members need to utilize local resources within their disposal as a means of sustaining projects. 78.8 % of the respondents (Table 2) confirmed that uneven work load among staff challenges project sustainability, which is in conformity with Vamos et al assertion that; uneven workload led to insufficient service provision, delayed job completion, and overtly contribute to workers burn out, thereby hindering sustainability [13]. To address the effect of uneven workload, adequate human capital is a necessity for effective job delivery.

Project sustainability thrived on resources availability for qualitative service provision and maintenance, this claim was in line with La Gargasson et al. [14] findings that insufficient financial resources encountered during introduction of new vaccines found in his study, hampered appropriate cold-chain maintenance and distribution of the vaccine. Also, Rashed et al. [15] cites inadequate resources as a challenge confronted by most health project hindering its sustainability, as such, sufficient resources are central to project maintenance and continuity. Therefore, working with available resources sustains projects beyond donor era; programs tailored on available resources address local needs in sustainable fashion as supported by 86.5% of the respondents (Table 3). Aubel & Samba-Ndure [16] found that projects built on available resources, produce sustainable outcomes. Their study reported that engagement of Traditional Birth Attendance (TBAs) and community leaders on women's nutritional education during pregnancy yield a good response. Logistic and other interventions like funding, capacity building, project monitoring and evaluation, are relevant to attaining program sustainability. This finding was in conformity with Humphries et al. [17] which state that infrastructure readiness, and functional project monitoring by advisory committee or board is an important intervention mechanism that maintains sustainability. With respect to CSOs dependency on donor funding impedes sustainability; the claim was accepted by 74.3% of the respondents (Table 3). This means that sustaining financial resources is the upper most priority of achieving project sustainability. Funding mechanisms of a project is cardinal to a country's health system strengthening pursuit. As such, CSOs projects not targeting a resilient health system, no matter how effective ["successful"] it may look, will always become non-sustainable in the long run. Therefore, projects should consider alternate financing sources for effective program sustainability, to avoid a pitfall of dependency on donor funding. Concerning the effect of operation friendly policies on program sustainability as reported by 87.8% of the respondents (Table 3), the finding here is in tandem with Ghiron et al [18] assertion that policies that address the need of the marginalized population are needed for sustainable project implementation. Operation friendly policy includes those that enhance accessibility to information, ease registration, enables freedom of association and expression among others.

## Conclusion

CSOs, respond to rising needs of the community they serve, and this provide them the opportunity of conducting activities that promote project sustainability, based on their functional ability. The activities include; information gathering, project monitoring and dialogue with stakeholders on issues bothering project sustainability. The study shows that right policies among other factors enhance CSOs efforts of achieving sustainability; as such project sustainability requires operation friendly policy, understanding of project pain point, available resources (human, money, and machinery), community participation, internal funding mechanism and partnership among other attributes. Indeed, CSOs engagement in partnership with diverse sectors in health system introduced sustainable approaches like community participation and utilization of available local resources, and as such, it is essential for CSOs to work in collaboration with private institutions, communities and governments, to sustain health projects, employing multi-sector approach reflective of Public Private Partnerships (PPPs). Succinctly, CSOs approach to health project sustainability should defy traditional approach of one sector disease prevention and control mechanism. As such, community-based health projects should be participatory and inclusive among the CSOs, government and the community; such approaches enhance efficiency and effectiveness of sustainable innovations that ensure project continuity.

### What is known about this topic

- Health project sustainability depends on financial and technical capacity for proper operation;
- Donor organization heavily influences their resource direction irrespective of prioritized needs;
- Practically, most health projects received almost no attention in post-donor era, indicating a blatant lack of concern on project sustainability issues.

### What this study adds

- Partnership arrangement between Civil Society Organizations (CSOs), government and community members or between CSOs and government enhance project sustainability;
- Local health project sustainability is achievable through Community participation;
- Adequate trained personnel are required to counter the effect of uneven workload.

## Competing interests

The authors declare no competing interests.
